# Effect of high-intensity interval training on cardiorespiratory fitness, physical activity and body composition in people with schizophrenia: a randomized controlled trial

**DOI:** 10.1186/s12888-020-02827-2

**Published:** 2020-08-27

**Authors:** Eivind Andersen, Gry Bang-Kittilsen, Therese Torgersen Bigseth, Jens Egeland, Tom Langerud Holmen, Egil Wilhelm Martinsen, Trine Stensrud, John Abel Engh

**Affiliations:** 1 Faculty of Humanities, Sports and Educational Science, University of South-Eastern Norway, PO box 235, 3603 Kongsberg, Horten Norway; 2 Division of Mental Health and Addiction, Vestfold Hospital Trust, Tønsberg, Norway; 3 Department of Psychology, University of Oslo, Oslo, Norway; 4 Clinic Mental Health and Addiction, Oslo University Hospital, Oslo, Norway; 5 Institute of Clinical Medicine, University of Oslo, Oslo, Norway; 6 Department of Sports Medicine, Norwegian School of Sports Sciences, Oslo, Norway

**Keywords:** Schizophrenia, RCT, Exercise, Maximal oxygen uptake, Physical activity

## Abstract

**Background:**

Exercise may improve cardiorespiratory fitness in people with schizophrenia, however, possible condition-specific cardiorespiratory disadvantages, a scarcity of methodologically sound studies, and conflicting results raise questions about the effect of exercise on maximal oxygen uptake (VO_2max_) in this group. The primary aim of this study, therefore, was to investigate the effect of high-intensity interval training on VO_2max_ in people with schizophrenia. Second, we sought to determine whether the intervention would have an effect on general physical activity (PA) level and body composition.

**Methods:**

Eighty-two patients with schizophrenia were randomly assigned to supervised high-intensity interval training or computer gaming skills training, performed twice a week for 12 weeks. Oxygen uptake was measured directly, during a maximum exercise session on a treadmill. PA level were assessed using ActiGraph accelerometer, and body composition was assessed by bioelectrical impedance. Differences between groups were assessed by analysis of variance using a univariate general linear model.

**Results:**

There were no significant differences between the groups on any of the cardiorespiratory variables neither at baseline nor after the program. There were also no significant within-group differences in any of the cardiorespiratory fitness variables between the baseline and post-program time points, despite that 61% of the participants performing high-intensity interval training showed a significant increase in workload on the treadmill. However, 47% of the participants in the high-intensity interval training group had a ≥ 5% increase in VO_2max_. Participants supervised by mental health care providers with PA competence (e.g. rehabilitation center staff, sport scientist, physical trainer) had a much larger increase in VO_2max_ compared to participants supervised by mental health workers without such competence, and when adding PA competence to the model, the intervention group increased VO_2max_ significantly compared to the comparison group. The intervention had no significant effect on PA level or body composition.

**Conclusions:**

The intervention did not improve VO_2max_, PA level or body composition but succeeded in increasing workload on the treadmill. With regard to VO_2max_, approximately half of the patients may be considered responders.

**Trial registration:**

ClinicalTrials.gov; NCT02205684, registered July 2014,

## Background

The effects of schizophrenia on years of potential life lost and life expectancy are substantial [1]. People with schizophrenia have an elevated risk of developing cardiovascular diseases (CVD) and a significantly increased risk of death from CVD compared to the general population, which may, to a large extent, explain the shortened life expectancy in this patient group [2–4]. The prevalence discrepancy in somatic disorders, such as CVD [2] and type 2 diabetes (T2D) [5, 6], between people with schizophrenia and the general population, is not fully understood. It is likely due to a complex interplay among many factors, such as the metabolic side effects of antipsychotics (i.e., leading to overweight) [7], tobacco smoking [8], alcohol misuse [9], poor dietary habits [10], low help-seeking behavior [11] and a possible genetic susceptibility to cardiovascular disease in people with schizophrenia [12, 13]. Additionally, low levels of moderate and vigorous intensity physical activity (MVPA) [14–16], excessive amounts of sedentary time [16, 17] (defined as any waking behavior characterized by an energy expenditure ≤1.5 metabolic equivalents while in a seated or reclining posture [18]), and poor cardiorespiratory fitness (CRF) levels [15, 19], have been recognized as potentially important factors for the high prevalence of somatic disorders [20–22].

In the general population, CRF levels have been linked to both genetic [23] and behavioral factors, including physical activity (PA) [24] and exercise [25]. The observed low PA level [15] and lack of exercise in people with schizophrenia may, therefore, be important factors explaining the poor CRF level. However, the picture may be more complex in this patient group. It has been proposed that, in addition to adverse metabolic effects, antipsychotic drugs also have adverse effects on the cardiovascular system (i.e., antagonistic activity at alpha-adrenergic receptors) [26–28]. It is also proposed that a high prevalence of chronotropic incompetence (attenuated heart rate response to exercise) [26, 29], reduced activity of the efferent vagal system [30] and mitochondrial dysfunction [31, 32] may be condition-specific factors related to their CRF level.

Because people with schizophrenia in general have low CRF level, which is closely linked to CVD and mortality, supporting this patient group to exercise and thereby increasing their CRF level seems appropriate. However, the abovementioned cardiovascular disadvantages may potentially negatively impact the effect of exercise on the cardiorespiratory system. Although one meta-analysis [33] and two systematic reviews [19, 34] all concluded that exercise significantly improves CRF levels in schizophrenia, most of the included studies had methodological shortcomings. For example, of the eight studies included in these papers, four did not include a control group [35–38], and one was nonrandomized [39]. Furthermore, the number of participants was generally very low, ranging from five to 16 in the exercise group. Finally, none of the studies controlled for physical effort using the respiratory exchange ratio (RER) value or any other possible confounder. Although criteria for what constitutes a valid CRF test were often given, it was not reported how many participants passed the criteria for a valid test. The only study with a relatively large sample size and a relatively rigorous design and analysis found a significant difference between the groups, but this was due to a decline in the CRF of the control group participants rather than increased CRF in the participants of the exercise group [40].

The positive effect of exercise, especially high-intensity interval training (HIIT), on CRF and health is well established in studies of the general population [41] and across several diseases-specific states [42]. Given the abovementioned possible cardiorespiratory disadvantages (e.g. chronotropic incompetence and mitochondrial dysfunction) in people with schizophrenia and conflicting results from the few studies conducted, the effect of exercise on CRF in this patient group is unclear. The primary aim of this study, therefore, was to investigate the effect of HIIT on CRF in people with schizophrenia. Second, we aimed to test whether partaking in an HIIT exercise regime in combination with applying key constructs from social cognitive theory (e.g., self-efficacy and social support for PA) would have an effect on the general PA level; third, we sought to test whether the intervention would have secondary effects on body composition.

## Methods

### Study design

This study is based on data from the Effects of Physical Activity in Psychosis study (EPHAPS) (ClinicalTrials.gov, registration number NCT02205684). The study was approved by the Regional Ethics Committee for Medical Research (2014/372). The methods used in EPHAPS have been described in detail elsewhere [43]. Briefly, the study was designed as a randomized, controlled, parallel-group, observer-blinded clinical trial with the aim of investigating the effects of a supervised high-intensity interval training program on maximal oxygen uptake (VO_2max_), cognitive function and psychiatric symptom load in outpatients with schizophrenia.

### Recruitment and participants

**Fig. 1:**
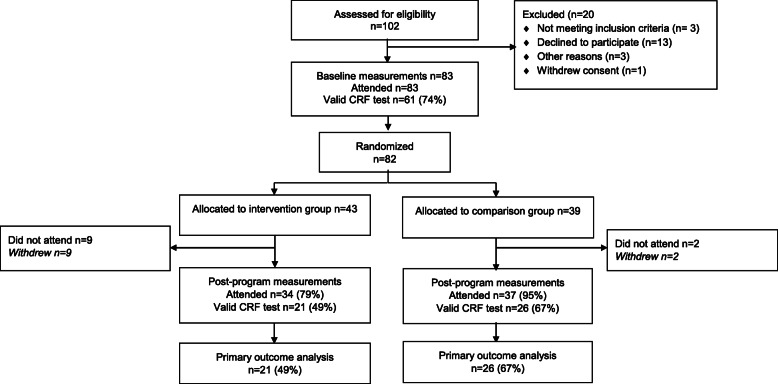
EPHAPS trial flowchart

Participants were recruited from August 2014 through May 2017 from catchment-area-based and publicly funded outpatient psychiatric clinics in Vestfold County, Norway. Patients aged 18–67 years old, IQ ≥ 70, who understood and spoke a Scandinavian language and fulfilled the criteria in The Diagnostic and Statistical Manual of Mental Disorders (5th ed.; DSM-5) [44] for schizophrenia spectrum disorder (schizophrenia, schizoaffective disorder, and schizophreniform disorder) were eligible for the study. Patients who were pregnant or had physical contraindications to exercise were excluded. Initial information about the study was given to eligible patients by clinical staff in the outpatient clinic or in primary health services. Written consent was obtained from those eligible patients who understood the nature of the research and were willing to participate. Eighty-three participants signed the informed consent form and were included in the study. One participant withdrew from the study after baseline testing but before randomization. A scheme of the flow of participants through the trial is presented in Fig. 1.

### Randomization and stratification

After the baseline assessments, the participants were randomly assigned to either the HIIT group (intervention group) or the computer gaming skills group (comparison group). A computerized random number generator was utilized to produce the allocation sequence. Equal distribution of CRF between the two groups of participants was ensured by stratification around the expected median of baseline VO_2max_ scores. By varying the size of the stratification blocks, we kept each treatment assignment unpredictable. A designated project coordinator, who was not involved in recruitment or measurement sessions, administered the group assignments using the method of concealed envelopes. The randomization process was performed at a site remote from where the intervention took place.

### Interventions

The HIIT sessions and the computer gaming took place at different locations and the participants in the two groups did not have any contact with each other. The same personnel, which were employed in the outpatient clinics and dedicated to the project throughout the intervention period, carried out supervision of the participants in both groups. The time spent with activities was the same in both groups, as was the time spent with the intervention staff. A specific time schedule was followed by the staff to ensure that each session in the exercise and comparison group lasted 45 min. In addition to the sessions, most of the participants in both groups were accompanied by a member of the intervention staff during transportation to and from the sessions. Both groups also received treatment as usual.

The exercise sessions consisted of supervised walking/running on a treadmill twice a week for 12 weeks. The exercise sessions were led by two teams each consisting of two persons. The two different teams had different background and experience with regard to exercise and physical activity. Half of the participants were supervised by mental health care providers with PA competence (rehabilitation center staff with relevant PA education), and the other half was led by mental health professionals without PA competence. Each session had the following structure: an 8-min warm up, followed by 4 × 4 min intervals at 85–95% of maximum heart rate, with active pauses consisting of 3 min of walking/running at approximately 70% of maximum heart rate, and a 5 min cool-down period at the end. Heart rate was measured and controlled continuously throughout the exercise session by the intervention staff to ensure that the participants’ intervals and active pauses were performed with the desired intensity. Peak heart rate from the VO_2max_ test was used to calculate prescribed exercise intensity. Additionally, the intervention staff attempted to increase the participants’ motivation for PA. Although delivered in informal settings, during transportation, and before and after sessions, the motivational work was individualized, structured and theory-based. Key constructs from social cognitive theory [45] were applied: opportunities to perform PA, social support for PA, confidence to engage in PA, expected benefits and costs of performing PA, knowledge and skill to perform PA, and personal goal setting.

Participants in the comparison group took part in sessions in which they performed computer-simulated sports activities, such as tennis, baseball, golf and bowling, on a Nintendo Wii Sports console (Nintendo, Kyoto, Japan). Physiological responses (i.e., oxygen consumption, cardiac output, metabolic rate) to Nintendo Wii tennis and baseball have been shown to be lower than the response to brisk treadmill walking when tested in bouts of 10 min [46].

### Measurements

*Cardiorespiratory fitness*, or maximal oxygen uptake (VO_2max_), was assessed through a maximum exercise test on a treadmill (Woodway, Würzburg, Germany), by physical activity professionals, before and after the 12-week intervention. We used a modified Balke protocol [47], where speed was held constant at 5 km∙h^− 1^ and the inclination angle was increased by one degree every minute until exhaustion within 6–12 min. Gas exchange was continuously sampled in a mixing chamber every 30 s by having the subjects breathe into a Hans Rudolph two-way breathing valve (2700 series, Hans Rudolph Inc., Kansas City, USA). The breathing valve was connected to a Jaeger Oxycon Pro (Erich Jaeger GmbH, Hoechberg, Germany), which was used to analyze the oxygen and carbon dioxide content. The Jaeger Oxycon Pro has been found to be both valid and reliable [48]. The test result was included in the current study if the respiratory exchange ratio (RER) (i.e., physical strain) was ≥1.00 [49]. Heart rate was recorded with an RCX3 heart monitor from Polar (Polar Electro Oy, Kempele, Finland). The oxygen pulse (ml∙beat^− 1^) was calculated by dividing VO_2max_ (mL∙min^− 1^) by the maximal heart rate (beats∙ min^− 1^) and used as an estimate of the heart’s stroke volume.

*Pulmonary function* was measured by maximal expiratory flow volume curves (MasterScreen Pneumo spirometer; CareFusion, Hochberg, Germany) according to current guidelines set by the American Thoracic Society and The European Respiratory Society in 2005 [50]. The items measured were forced vital capacity (FVC), forced expiratory volume in 1 s (FEV_1_), FEV_1_/FVC and maximal expiratory flow at 50% of FVC (MEF 50%). Maximal voluntary ventilation (MVV) was estimated as FEV_1_ multiplied by 35. In addition, breathing reserve (%) was calculated using the following equation: ([MVV - VE_max_]/MVV) × 100, where VE_max_ is the maximal minute ventilation during the exercise test. Predicted spirometry values were according to [51]. Obstructive lung disease was identified by FEV_1_/FVC < 0.7 [52].

*Physical activity* was assessed using the ActiGraph GT3X+ accelerometer (ActiGraph, LLC, Pensacola, FL, USA) in vertical axis collection mode. The participants were instructed to wear the accelerometer over the left hip while awake, except during water-based activities (e.g., showering, swimming), for four consecutive days. The epoch length (sample interval) was set to 10 s. All data were reintegrated into 60 s epochs and further processed and analyzed using specialized accelerometer analytical software (Kinesoft, version 3.3.80, Saskatchewan, Canada). Analyses were restricted to participants who wore the accelerometer for a minimum of 10 h per day [53] for at least two days (including one weekday and one weekend day) [54–56]. To identify different intensities of PA, we applied count thresholds corresponding to the energy cost of the given intensity, where sedentary time was defined as all activity < 100 counts per minute (CPM) [57], a threshold that corresponds with sitting, reclining, or lying down. Light intensity PA was defined as 100–2019 CPM, moderate as 2020–5998 CPM, and vigorous as CPM ≥ 5999 [58].

*Body composition* was estimated barefooted in light clothing by bioelectrical impedance (MC-980 MA; Tanita Corp., Tokyo, Japan). Assessment of body composition was performed around noon, approximately three hours after a light breakfast, at both time points. Weight was measured to the nearest 0.5 kg in light clothing and no shoes using a SECA electronic scale (SECA model 767; Hamburg, Germany). Height was measured without shoes on a portable stadiometer (Harpenden; Holtain, Crymych, Wales) and rounded to the nearest 0.5 cm.

Data on education, employment, marital status and smoking were obtained through a clinical interview and the use of hospital charts. Positive and negative psychotic symptom levels, as well as the total general symptom level were assessed with the Positive and Negative Syndrome Scale (PANSS) [59]. Information on current medication was acquired through patients’ interviews and the use of medical records and information from the general practitioners. Defined daily doses (DDD) were calculated in accordance with guidelines from the World Health Organization Collaborating Center for Drug Statistics Methodology (http://www.whocc.no/atcdd). The DDD is the assumed average maintenance dose per day for a drug used for its main indication on adults and provide a fixed unit of measurement independent of dosage form, providing a rough estimate of pharmaceutical drugs consumption.

### Statistical analysis

All statistical analyses were performed using SPSS (Statistical Package for the Social Sciences for Windows, version 24, IBM, Inc., Chicago, IL, USA). Descriptive data are presented as proportions, means, and standard deviations (SDs) with 95% confidence intervals (CIs) where appropriate. Within- and between-group differences in interval data were evaluated by *t-*tests (independent *t-*tests and paired *t-*tests). Differences between groups were assessed by analysis of variance using a univariate general linear model. Baseline CRF variables (dependent variables) were adjusted for RER, age and sex (covariates). Post-program CRF variables were adjusted for post-program RER, age and sex. After the completion of our trial, Firth et al. (2017) showed in a meta-analysis that exercise studies that were supervised by PA professionals (e.g. rehabilitation center staff, sport scientist, physical trainer) were more effective [60]. Our study was not designed or powered to look at staff competence, but present explorative analysis of time x group x staff PA competence, using a three-way repeated measures ANOVA. A univariate general linear model was used to assess differences between the groups PA level (CPM, sedentary time, light intensity PA, moderate intensity PA, vigorous intensity PA and steps per day). Accelerometer wear time was considered a potential confounder and included in the analysis. Differences between the groups in terms of education level, employment and marital status were assessed using chi-squared tests. Treadmill speed and/or inclination on interval number three in weeks four and 12 was analyzed to estimate progress or work capacity. For work capacity, week four was set as baseline measure to avoid a learning effect on the treadmill. All significance tests were two-sided.

## Results

Table 1 displays the relevant demographic and clinical information for both groups. Except for three participants, all were using antipsychotic medication. Sixty-two participants used second-generation antipsychotics (SGA) and six used first-generation antipsychotics (FGA). Of the 25 participants receiving ≥ two different antipsychotic drugs, 11 received a combination of FGA and SGA. There were no significant differences between groups with respect to type of antipsychotic medication and the main metabolic risk profile of these drugs [61, 62]. Defined daily doses of antipsychotics was slightly higher in the intervention group compared to the comparison group (mean diff 0.4, 95% CI 0.0, 0.8; *P* = 0.04). There were no other statistical differences between the groups at baseline. In addition, regular antidepressants, mood stabilizing medication and anxiolytics were received by 19, 12 and 11 participants, respectively.

**Table 1:** Baseline sociodemographic and clinical characteristics for both groups

Characteristics	Intervention group (*n* = 43)	Comparison group (*n* = 39)
Female (%)	39	38
Age (years)	36 (14)	37 (13)
PANSS		
Total	68 (16)	63 (16)
Negative	19 (7)	17 (6)
Positive	15 (5)	14 (5)
Duration of illness (years)	15 (11)	15 (13)
Antipsychotics (DDD)*	1.8 (1.0)	1.3 (0.7)
Smokers (%)	62	71
Education level (%)		
Primary school	44	41
High school	37	28
College/university	18	30
Employed at least 50% of full time (%)	11	10
Married or living with a partner (%)	14	13

Presented values are the mean ± SD if not specified otherwise. DDD, defined daily doses. PANSS; Positive and Negative Syndrome Scale. * Significant difference between groups (*P* ≤ 0.05)

Of the 43 participants randomized to HIIT, 34 (79%) attended the post-program measurements and 21 (49%) had a valid VO_2max_ test (Fig. 1). Of the 39 participants randomized to the comparison group, 37 (95%) attended the post-program measurements and 26 (67%) had a valid VO_2max_ test (Fig. 1).

There were no differences between groups in any of the cardiorespiratory variables either at baseline or after the program (Table 2). There were no within-group differences in any of the cardiorespiratory fitness variables between the baseline and post-program time points. However, 47% of the participants in the intervention group and 27% in the comparison group had a ≥ 5% increase in VO_2max_ (mL∙kg^− 1^∙min^− 1^). We classified participants with ≥1 metabolic equivalent (MET) increase in VO_2max_ (mL∙kg^− 1^∙min^− 1^) as responders [63]. The responders had, adjusted for gender, significantly lower VO_2max_ at baseline (mean diff. -8.4 mL∙kg^− 1^∙min^− 1^, 95% CI -15, − 1.4; *P* = 0.02) were 7.7 (14, 0.4; *P* = 0.03) years older and had higher BMI (4.0 kg∙m^− 2^, 0.5, 7.6; P = 0.02), waist circumference (11 cm 21, 1.4; P = 0.02) and visceral fat mass (5.5 kg, 8.5, 2.6; P < 0.0001) than non-responders (<5% increase in VO_2max_ (mL∙kg^− 1^∙min^− 1^). No other differences were found between responders and non-responders, including exercise performance (number of sessions, number of intervals, number of intervals above 85% of maximum heart rate).

**Table 2:** Between-group difference in cardiorespiratory responses to maximal exercise at baseline and post-program

		Intervention group		Comparison group				
		N	Mean (SD)	N	Mean (SD)	Mean diff	95% CI	P
VO_2max_ (L·min^− 1^)	Baseline	34	2.7 (0.7)	27	2.5 (0.9)	0.05	0.2, −0.3	0.7
	Post-program	20	2.7 (0.9)	26	2.6 (0.9)	0.1	0.3, − 0.1	0.3
VO_2max_ (mL∙kg^−1^∙min^−1^)	Baseline	34	32.1 (11)	27	31.1 (11)	0.9	3.6, −5.5	0.7
	Post-program	21	30.8 (11)	26	31.1 (11)	−1.8	−4.4, 0.8	0.17
VO_2max_ (mL∙kgFFM^−1^∙min^− 1^)	Baseline	34	44.8 (12)	27	42.6 (12)	0.2	5.5, −4.9	0.9
	Post-program	20	43.4 (12)	26	43.2 (12)	1.7	5.6, −2.1	0.3
RER (VCO_2_/VO_2_)	Baseline	34	1.09 (0.05)	27	1.09 (0.06)	0.00	0.02, −0.04	0.5
	Post-program	21	1.10 (0.07)	26	1.08 (0.06)	0.01	0.05, −0.02	0.3
Maximal heart rate (beats·min^− 1^)	Baseline	34	174 (22)	27	170 (17)	0.4	8, −7	0.9
	Post-program	20	173 (20)	26	169 (13)	1.8	5.4, −1.7	0.3
Ventilation (L∙min^− 1^)	Baseline	29	94 (29)	22	84 (30)	2.0	−16, 12	0.7
	Post-program	21	88 (27)	24	85 (25)	3.8	−13, 5	0.4
Oxygen pulse (mL∙beats^− 1^)	Baseline	34	15.5 (3.9)	27	14.5 (4.7)	0.4	2.3, −1.3	0.5
	Post-program	19	16.0 (4.3)	25	15.2 (5.1)	0.6	2.0, −0.7	0.3
Resting heart rate (beats∙min^− 1^)	Baseline	34	75 (12)	27	75 (16)	0.9	8, −6	0.7
	Post-program	21	75 (13)	24	72 (14)	0.4	7, −6	0.8
Heart rate reserve (beats∙min^−1^)	Baseline	34	98 (25)	27	94 (26)	0.5	10, −11	0.9
	Post-program	19	97 (21)	23	97 (23)	1.2	8.8, −6.4	0.7

Presented values are the mean ± SD. VO_2max_, peak oxygen uptake. FFM, fat-free mass. RER, respiratory exchange ratio. CI, confidence interval

The HIIT participants led by mental health workers with PA competence had a significantly larger increase in CRF (2.4 mL∙kg^− 1^∙min^− 1^ ± 4.5) compared to the HIIT participants led by mental health workers without such competence (− 2.2 mL∙kg^− 1^∙min^− 1^ ± 4.7), with a mean difference of 4.7 mL∙kg^− 1^∙min^− 1^ (95% CI = 1.2, 8,1; *P* = 0.010). There were no significant differences in number of sessions, number of intervals or number of intervals performed at ≥85% of maximal heart rate between the two HIIT groups. So, when controlling for staff PA competence there was a significant difference in CRF between the intervention group and the comparison group (*F* [1, 60] = 11.4, *p* = 0.001; Wilk’s Λ = 0.84, partial ƞ2 = 0.16).

The average number of sessions conducted was 18.1 ± 4.3 and 19.2 ± 2.0 (mean diff − 1.1, 95% CI -2.6, 0.4; *P* = 0.16) in the intervention and comparison groups, respectively, and participation ranged from 6 to 24 sessions in the intervention group and 15 to 24 in the comparison group. In the intervention group, 83% of the participants attended ≥60% of the sessions, and 54% attended ≥80% of the sessions. In the comparison group, 100 and 70% attended ≥60% and ≥ 80% of the sessions, respectively. There was no significant difference in any of the cardiorespiratory variables after we excluded those who participated in either <60% or <80% of the sessions. Of a possible total of 96 intervals, the exercise group conducted, on average, 69 ± 19 intervals (range 16 to 96). 39.9% of the intervals was performed ≥85% of maximal heart rate. There was no significant correlation between VO_2max_ and the number of intervals performed. However, there was a significant correlation (r = 0.43; *P* = 0.14) between number of intervals performed at ≥85% of maximum heart rate and change in VO_2max_.

Participants in the exercise group showed significant improvements in work capacity, shown by both improved treadmill running speed and inclination from week four to week 12 (Table 3). Sixty-one percent improved running speed and/or inclination. Eighty-six percent of the responders (≥ 1MET increase in VO_2max_), compared to 53% of the non-responders (< 1 MET increase in VO_2max_) improved work capacity (*P* = 0.50). Ninety percent of the participants achieved ≥85% of maximum heart rate during both exercise sessions.

**Table 3:** Work capacity measured at interval number three in weeks 4 and 12 in the group performing high-intensity interval training

		Mean (SD)	Mean diff	95% CI	P
Treadmill speed (km/h)	Week 4	6.1 (2.2)	0.5	0.2, 0.9	0.002
	Week 12	6.7 (2.8)			
Treadmill inclination (%)	Week 4	4.1 (2.8)	1.6	0.2, 2.9	0.02
	Week 12	5.7 (5.1)			
Heart rate (beats/min)	Week 4	155 (21)	3.9	0.8, 7.1	0.01
	Week 12	159 (22)			

*N* = 36. Presented values are the mean ± SD. CI, confidence interval

There were no differences in pulmonary function between groups or within groups at either time point (Table 4). Baseline VO_2max_ (mL∙kg^− 1^∙min^− 1^) had a moderate correlation with FVC (r = 0.31, *P* = 0.01), FEV_1_ (r = 0.35, P < 0.001) and MEF_50_ (r = 0.40, P < 0.01). Twelve percent had < 80% of predicted FEV_1_, and 10.5% scored below 70% on the FEV_1_/FVC ratio (FEV_1_%). Thirty percent had a low breathing reserve (< 15%), whereas 32% had a high breathing reserve (> 40%) that may suggest pulmonary or cardiovascular limitations, respectively, for exercise performance [64].

**Table 4:** Between-group differences in pulmonary function at baseline and post-program

		Intervention group		Comparison group				
		N	Mean (SD)	N	Mean (SD)	Mean diff	95% CI	P
FVC (L)	Baseline	43	4.4 (1.3)	39	4.2 (1.1)	0.2	−0.3, 0.7	0.4
	Post-program	32	4.3 (1.3)	37	4.2 (1.1)	0.0	−0.5, 0.6	0.7
FVC % pred (%)	Baseline		100 (20)		96 (15)	3.3	−4.9, 11.6	0.4
	Post-program		99 (17)		98 (14)	−1.3	−8.5, 5.9	0.7
FEV_1_ (L)	Baseline	43	3.4 (1.0)	39	3.3 (1.0)	0.1	−0.3, 0.6	0.5
	Post-program	32	3.3 (1.0)	37	3.1 (0.9)	0.1	−0.3, 0.6	0.4
FEV_1_ pred (%)	Baseline		93 (16)		90 (19)	2.7	−56, 11.1	0.5
	Post-program		93 (18)		89 (17)	1.8	−5.8, 9.6	0.6
FEV_1_/FVC (%)	Baseline	42	77 (11)	37	77 (10)	−0.1	−4.9, 4.6	0.9
	Post-program	32	78 (8)	37	75 (11)	2.5	−2.2, 7.3	0.2
FEV_1_/FVC pred (%)	Baseline		95 (13)		95 (12)	−0.3	−6.2, 5.6	0.9
	Post-program		96 (9)		93 (14)	3.7	− 2.2, 9.7	0.2
MEF_50_ (L· s^−1^)	Baseline	42	3.9 (1.5)	37	3.9 (1.4)	0.0	−0.5, 0.7	0.8
	Post-program	32	3.8 (1.6)	37	3.4 (1.3)	0.4	−0.3, 1.1	0.2
MEF_50_ pred (%)	Baseline		78 (25)		78 (25)	0.2	−11.6, 12.1	0.9
	Post-program		78 (27)		70 (25)	8.0	−5.0, 21	0.2
MVV (L)	Baseline	40	120 (37)	37	114 (36)	5.7	−11, 22	0.5
	Post-program	32	118 (37)	35	110 (31)	7.8	−9, 24	0.3
BR (%)	Baseline	37	26 (18)	32	22 (26)	4.2	−7, 15	0.4
	Post-program	25	21 (20)	27	20 (23)	0.8	−11, 13	0.8

Presented values are the mean ± SD. SD, standard deviation. CI, confidence interval. Pred, predicted. FVC, forced vital capacity. FEV_1,_ forced expiratory volume in 1 s. MEF 50%, maximal expiratory flow 50%. MVV, maximal voluntary ventilation. BR, breathing reserve

There were no significant differences between the groups in PA level or sedentary time at either time point (Table 5). No statistical changes were found within groups in any of the PA or sedentary variables. There were no associations between changes in VO_2max_ and any of the PA variables.

**Table 5:** Between-group differences in physical activity level and sedentary time at baseline and post-program

		Intervention group		Comparison group				
		N	Mean (SD)	N	Mean (SD)	Mean diff	95% CI	P
Number of valid days	Baseline	35	3.4 (1.0)	35	3.4 (1.2)	0.0	−0.5, 0.6	0.8
	Post-program	23	3.1 (1.1)	25	3.1 (1.0)	−0.0	− 0.6, 0.6	0.9
Wear time (min∙day^−1^)	Baseline	35	10.3 (2.9)	35	10.7 (3.1)	−0.3	−1.9, 1.1	0.6
	Post-program	23	10.6 (2.3)	25	10.8 (2.8)	−0.2	−1.7, 1.3	0.7
Total PA level (CPM)	Baseline	33	220 (91)	34	255 (157)	−35	−97, 26	0.25
	Post-program	22	246 (154)	25	281 (198)	−34	− 121, 52	0.4
Light PA (min∙day^− 1^)	Baseline	35	202 (90)	35	205 (91)	−2.6	−40, 34	0.8
	Post-program	23	226 (92)	25	199 (78)	27	−13, 68	0.18
Moderate PA (min∙day^− 1^)	Baseline	35	19 (12)	35	26 (23)	−7.0	−16, 2.6	0.15
	Post-program	23	24 (18)	25	21 (21)	2.7	−0.5, 11	0.5
Vigorous PA (min∙day^−1^)	Baseline	35	0.6 (2.1)	35	1.4 (3.7)	−0.8	−2.3, 0.7	0.3
	Post-program	23	1.9 (3.6)	25	2.1 (9.9)	−0.1	−2.7, 2.3	0.8
MVPA (min∙day^−1^)	Baseline	35	20 (12)	35	28 (24)	−7.8	−17, 2.0	0.11
	Post-program	23	26 (20)	25	23 (26)	2.7	−0.6, 11	0.5
Steps per day	Baseline	33	3984 (1519)	34	4532 (3262)	− 548	− 1713, 617	0.3
	Post-program	22	4568 (2509)	25	4620 (2825)	−52	− 1474, 1369	0.9
Sedentary time (hours∙day^−1^)	Baseline	35	8.2 (1.6)	35	8.2 (1.6)	−0.0	−0.7, 0.7	0.9
	Post-program	23	8.3 (1.6)	25	8.1 (1.6)	0.1	−0.8, 1.1	0.7

Presented values are the mean ± SD. PA, physical activity. CPM, counts per minute. MVPA, moderate to vigorous physical activity. CI, confidence interval

There were no significant differences in body composition between groups at either time point (Table 6). No statistically significant changes were found within groups in any of the body composition variables.

**Table 6:** Between-group difference in weight, BMI, waist circumference and body composition at baseline and post-program

		Intervention group		Comparison group				
		N	Mean (SD)	N	Mean (SD)	Mean diff	95% CI	P
Weight (kg)	Baseline	43	90.3 (21)	39	88.6 (24)	1.7	−8.6, 12	0.7
	Post-program	32	91.8 (21.3)	37	88.3 (25)	0.6	−2.2, 1.0	0.4
BMI (kg/m^2^)	Baseline	43	29.8 (5.8)	39	29.3 (6.3)	0.4	−2.2, 3.1	0.7
	Post-program	32	30.3 (5.8)	37	29.3 (6.7)	0.1	0.6, −0.4	0.6
Waist circumference (cm)	Baseline	41	105 (16)	37	104 (18)	0.6	7, −8	0.8
	Post-program	33	107 (15)	34	103 (18)	1.3	3, −0.7	0.19
Percent fat	Baseline	43	30.5 (8.1)	39	29.3 (10.6)	1.1	3, −5	0.5
	Post-program	32	30.7 (8.5)	37	29.7 (11.1)	0.0	0.9, −0.8	0.9
Fat mass (kg)	Baseline	43	28.1 (10.8)	39	27.5 (15.5)	0.6	5, −6	0.8
	Post-program	32	28.8 (11.2)	37	27.9 (16.1)	0.2	1.3, −0.8	0.6
Muscle mass (kg)	Baseline	43	59.1 (14.0)	39	58.0 (12.7)	1.0	4, −6	0.7
	Post-program	32	59.8 (13.2)	37	57.4 (12.8)	0.3	1.2, −0.4	0.3
Fat-free mass (kg)	Baseline	43	62.2 (14.7)	39	61.0 (13.2)	1.2	4, −7	0.7
	Post-program	32	62.9 (13.8)	37	60.4 (13.4)	0.2	1.1, −0.6	0.5
Visceral fat (kg)	Baseline	41	9.7 (4.8)	33	8.3 (5.0)	1.4	0.8, −3	0.2
	Post-program	30	9.4 (4.5)	36	9.2 (6.4)	0.1	0.6, −0.3	0.5

Presented values are the mean ± SD. BMI, body mass index. CI, confidence interval

## Discussion

This is the largest study to date investigating the effects of HIIT in schizophrenia, compared with with low intensity game-based exercise. We managed in the current study to recruit patients with schizophrenia into a highly physically demanding exercise program. Most of the participants adhered to the exercise protocol in terms of both the number and the intensity of the sessions. The intervention group did not significantly improve their VO_2max,_ and no significant difference between the groups was found after the program, even when we excluded those that did not adhere to the exercise protocol. However, approximately half of the participants responded satisfactory (increased VO_2max_ with ≥1 MET) to the intervention. The intervention group, especially the responders, did increase their work capacity, as shown by the increased workload on the treadmill (i.e. speed and/or inclination). When controlling for PA competence among intervention staff in explorative analysis, the intervention group significantly increased VO_2max_ compared to the comparison group. The intervention did not have any effect on the PA level, sedentary behavior or body composition of the participants.

Although there were no significant within- or between-group differences, almost half of the participants in the intervention group had 5 % or greater increase in VO_2max_. The participants who responded (i.e., with increased VO_2max_) to the exercise regime did not differ from the non-responders with respect to the quantity or quality of training conducted, but we did find a correlation between change in VO_2max_ and number of intervals performed at ≥85% of maximum heart rate. Importantly, the responders had a lower VO_2max_ and were heavier and older than the non-responder at baseline, and thus an especially important target group within this population. Our results may imply that people with schizophrenia can respond as expected to HIIT but that there may be more non-responders in this patient group than in the general population [65]. If our results hold true, we need to further investigate why some patients do not respond to HIIT to see if measures can be taken (e.g., medication) and perhaps to a larger extent treating underlying pulmonary and cardiovascular diseases affecting normal physiological adaptation to exercise.

Regarding the effect of HIIT on VO_2max_ in this patient group, the literature is not consistent. The EPHAPS study reinforces the uncertainty of whether this patient group as a whole responds as expected to this type of training. As mentioned in the introduction, there might be condition-specific and medication-related factors hindering their ability to adapt normally to HIIT and thus preventing the physiological adaptations necessary to increase VO_2max_.

One of the possible benefits of HIIT is an improvement of VO_2max_, and thus involves several organs. VO_2max_ is primarily limited by the ability of the cardiorespiratory system to deliver oxygen to the exercising muscle [66]. This means that, theoretically, one would need to make adaptations related to the heart’s stroke volume in order to increase VO_2max_. Thus, one possible explanation for the lack of effect on VO_2max_, although measured indirectly, might be the fact that the participants’ stroke volume did not increase. There might also be other plausible reasons for the lack of effect, such as the high prevalence of abnormal breathing reserve (indicating pulmonary or heart disease) and a relatively high prevalence of cardiovascular and respiratory disease. Furthermore, in a systematic review including 35 studies using population-based samples, 97 genes were found to predict VO_2max_ trainability, and lower responders had fewer positive response alleles than higher responders [67]. Thus, one could imagine that people with schizophrenia have fewer positive response alleles than other populations, or that potential epigenetic modifiers, such as medication, sleep, and body fat, influence gene expression and molecular function, thereby influencing VO_2max_ training response [67].

As discussed above, there might be several reasons for the lack of significant effect on VO_2max_. The most plausible reason however, may coincide with the exercise regime per se. The total exercise volume (number of sessions per week, number of intervals per session, intensity) and/or the number of participants adhering to the protocol might have been insufficient to obtain a significant effect on VO_2max_. The significant correlation between number of intervals performed ≥85% of maximal heart rate and improvements in VO_2max_ supports the view that the exercise intensity, on a group level, might have been too low on too many intervals. The exercise protocol in the current study, however, is widely used, with success, in different populations and should have been sufficient to obtain positive results [41, 42], moreover, most of the participants adhered to the strictly supervised sessions, both in terms of number of intervals and intensity. Furthermore, a recently published study on people with schizophrenia, using exactly the same exercise protocol, with fewer participants than in our study, did find a significant effect on VO_2max_ in the exercise group compared to a control group [68].

There was a markedly difference in response to the intervention with regard to CRF improvement within the HIIT group. The subgroup supervised by mental health care providers with PA competence had a significant increase in CRF, while the subgroup supervised by mental health care providers without such competence did not. We did not find any differences with regard to adherence to the exercise protocol between the two subgroups, but this may be due to lack of statistical power and/or validity of training diaries. These analyses were done post hoc, based on the findings by Firth et al. (2017), which found that the effect of exercise was larger among patients supervised by PA professionals [60]. Half of our participants were supervised by PA professionals and when adding PA competence to the model there was a significant increase in CRF in the intervention group compared to the comparison group. We do not have data to explain this result and can only speculate if our main results would have been different if all participants were supervised by health care providers with PA competence.

The intervention did not change the participants’ total PA level. Performing HIIT twice a week was probably enough of a challenge for the participants, and a “light-touch” PA component designed to increase motivation for PA was not grasped. Furthermore, the lack of improved body composition is in line with previous research [33]. An improvement in body composition would probably demand both an increased total PA level and an adapted diet [69].

The EPHAPS study has a number of strengths. First, we were able to recruit a large number of patients into a methodologically rigorous RCT involving a highly physical demanding exercise protocol, designed to increase VO_2max_, and a sound comparison condition. The broad recruitment strategies and wide inclusion criteria markedly strengthen the generalizability of the results. Second, each exercise session was carefully supervised by skilled personnel, who most likely contributed considerably to the number of patients who fulfilled the relatively demanding exercise requirements (i.e., number of sessions and intensity of intervals). Other major strengths of this study included direct measurement of VO_2max_ and stricter conditions than previous research for what constitutes a valid result, objective measurement of PA levels, and reliable measurement of body composition and pulmonary function. One major weakness affecting the power of the study was the high number of invalid VO_2max_ tests. Although most of the patients exerted maximum effort during the VO_2max_ test, many did not manage to achieve a respiratory exchange ratio of ≥1.00 and hence failed to attain a valid test result.

## Conclusions

The intervention did not improve maximal oxygen uptake, physical activity level or body composition but succeeded in increasing work capacity. With regard to maximal oxygen uptake, approximately half of the participants may be considered responders. Although most of the participants adhered to the exercise protocol, the correlation between number of intervals performed ≥85% of maximal heart rate with improvement in VO_2max_, leaves some uncertainty of whether an even stricter intensity-supervision would have yielded different results. Further investigation is needed to determine why so many did not respond to the exercise regime and to identify the consequences of non-response. HIIT is a demanding exercise protocol, especially for patients with severe mental disorders, who may benefit from supervision of health care providers with competence on physical activity rather than health care providers without such competence.

## Data Availability

Data can be accessed upon request.

## Abbreviations

CVD: cardiovascular disease
T2D: type 2 diabetes
MVPA: moderate- and vigorous intensity physical activity
CRF: cardiorespiratory fitness
PA: physical activity
RER: respiratory exchange ratio
HIIT: high-intensity interval training
EPHAPS: effects of physical activity in psychosis study
VO_2max_: maximal oxygen uptake
FVC: forced vital capacity
FEV_1_: forced expiratory volume in 1 s
MEF 50%: maximal expiratory flow 50%
MVV: maximal voluntary ventilation
DDD: defined daily doses
SGA: second generation antipsychotics
FGA: first generation antipsychotics
MET: metabolic equivalent
PANSS: positive and negative syndrome scale
FFM: fat free mass
CI: confidence interval
SD: standard deviation
BR: breathing reserve
CPM: counts per minute
BMI: body mass index
